# Psychopathology from childhood to adolescence in children with and without Mild Intellectual Disability (MID): a comparison across three MID operationalizations

**DOI:** 10.1007/s00787-026-03106-3

**Published:** 2026-06-22

**Authors:** Dogukan Koc, Maurits Masselink, Stefania Barzeva, Daan H. G. Hulsmans, Nanda N. J. Rommelse, Roy Otten, Catharina A. Hartman, Pauline W. Jansen

**Affiliations:** 1 Department of Child and Adolescent Psychiatry/Psychology, Erasmus MC, University Medical Centre Rotterdam – Sophia, Rotterdam, the Netherlands; 2 Generation R Study Group, Erasmus University Medical Centre, Erasmus University Rotterdam, Rotterdam, the Netherlands; 3 Behavioral Science Institute, Radboud University, Nijmegen, The Netherlands; 4 Department of Psychiatry, Radboud University Medical Center, Nijmegen, The Netherlands; 5 Interdisciplinary Center Psychopathology and Emotion Regulation (ICPE), Department of Psychiatry, University of Groningen, University Medical Center Groningen, Groningen, The Netherlands; 6 Faculty of Behavioural and Social Sciences, University of Groningen, Groningen, The Netherlands; 7 Karakter Child and Adolescent Psychiatry University Centre, Nijmegen , The Netherlands; 8 Developmental Psychology, Utrecht University, Utrecht, the Netherlands; 9 Department of Psychology, Education and Child Studies, Erasmus School of Social and Behavioral Sciences, Erasmus University Rotterdam, Rotterdam, the Netherlands; 10 Department of Child and Adolescent Psychiatry/Psychology, Erasmus MC – Sophia Children’s Hospital, Postbus 2060, 3000 CB Rotterdam, The Netherlands

**Keywords:** Mild intellectual disability, Psychopathology, DSM-5, Longitudinal studies

## Abstract

The DSM-5(-TR) expanded the definition of Mild Intellectual Disability (MID) to include adaptive functioning (AF) alongside intelligence quotient (IQ). In this population-based study, we examined behavioural and emotional problems in children with MID and matched controls from early childhood to adolescence. We evaluated how three operationalizations of MID, varying by IQ and AF, influence outcomes. Cognitive assessments and parent-reported problems from 4,643 children from 1.5 to 15 years (T1 to T5) in the Generation R Study were used. MID was defined as: (1) IQ ≤ 75; (2) IQ ≤ 75 with impairments in AF; (3) IQ ≤ 85 with impairments in AF. Propensity score matching ensured comparable MID and non-MID groups. Linear mixed-effects models were employed to assess multiple domains of psychopathology over time. Across all MID definitions, children with MID exhibited elevated behavioural and emotional problems compared to matched controls. Children meeting the criteria for MID3, the broadest definition, showed the highest problem levels, with elevated ADHD (β = 0.37, *p*FDR < 0.001), affective (β = 0.25, *p*FDR < 0.001), and autistic problem scores (β = 0.25, *p*FDR < 0.001) at baseline, with differences widening over time. Children with MID1 and MID2 also displayed unfavourable ADHD trajectories, although no baseline differences were observed. The findings suggest the extent of mental health problems depends on how MID is operationalized and suggest that psychopathology may be particularly present in children with borderline intellectual disability, 75 ≤ IQ ≤ 85.

**Supplementary Information:**

The online version contains supplementary material available at https://doi.org/10.1007/s00787-026-03106-3.

## Introduction

Approximately 2–3% of children and adolescents have an Intellectual Disability (ID), defined by significant limitations in intellectual and adaptive functioning (AF) relative to developmental expectations [1–3]. Intellectual functioning reflects general cognitive abilities such as reasoning, problem-solving, and learning, whereas AF encompasses the conceptual, social, and practical skills needed for everyday life. The severity of ID is classified as mild, moderate, severe, or profound, with about 85% of affected children falling within the mild range (MID), which is the focus of this study.

Children with ID show a markedly higher prevalence of mental health problems than their peers without ID, with rates of 30–50% compared to 13–16% in the general population [3–5]. Elevated internalizing (e.g., anxiety, depression) and externalizing (e.g., aggression, hyperactivity) symptoms emerge early in childhood [6–8] and often persist across development [6]. In the general population, externalizing behaviours decline from preschool to adolescence [9, 10], whereas internalizing problems stabilize or increase, particularly among girls [9, 11–13]. Children with ID, who face challenges in adaptive functioning, may have fewer resources to manage developmental demands, leading to slower reductions in externalizing and greater increases in internalizing symptoms over time. However, longitudinal evidence remains inconsistent: some studies report a sharper rise in internalizing and slower decline in externalizing problems [7], while others find similar or even faster decreases among children with ID [6, 8]. Such discrepancies likely reflect differences in informants, developmental periods, and especially in how ID has been operationalized across studies.

In this study, we investigated associations between ID status and psychopathology using three operationalizations of ID, which differ in their focus on IQ and AF. These operationalizations reflect both historical reliance on IQ scores and the more recent emphasis on AF introduced in the DSM-5. Importantly, this conceptual shift is not unique to DSM-based frameworks: ICD-11 similarly defines intellectual developmental disorders based on impairments in both intellectual and adaptive functioning, supporting the broader relevance of the present approach across diagnostic systems [14]. In line with common clinical and research practice, our first operationalization (MID1) is based on IQ alone and defined as an IQ score of ≤ 75 (70 + a 5-point measurement error margin). That is, even though both intellectual functioning (reflected by an IQ score) and clinical judgment [4]) and AF have been included in the criteria for (M)ID across subsequent versions of the Diagnostic and Statistical Manual of Mental Disorders (DSM) and similar psychopathology classification systems in the past decades, in practice, IQ tended to be regarded as the most important criterion for identifying ID. This means that AF was often not considered, and a MID diagnosis was based solely on an IQ score between 50 and 70 [15–17]. In keeping with this practice, MID1 is based solely on IQ. With the introduction of the DSM-5, the emphasis in diagnostic criteria shifted towards AF in contrast to previous editions. The severity of ID is currently determined by the degree of impairment in AF. The DSM-5-TR further clarifies that a MID diagnosis is not appropriate when IQ scores are substantially higher than 65–75. Our second operationalization (MID2) aligns with the DSM-5-TR definition for MID: IQ ≤ 75 combined with impairments in at least one of the three AF domains (conceptual, social, and practical). Our third conceptualization (MID3) additionally included individuals with IQ scores between 75 and 85 who also show impairments in at least one of the three AF. This broader conceptualization acknowledges that individuals in this range often require support [18]. In line with this perspective, the Netherlands uses a broader definition of MID, extending IQ to 85 to include this group.

This study examined the developmental trajectories of psychopathology from early childhood to adolescence among children with and without MID within a population-based cohort. Given that definitions of MID vary across research and clinical practice, we also evaluated whether the choice of operationalization influenced observed associations. Beyond internalizing, externalizing, and neurodevelopmental domains, we included less frequently studied problems, such as sleep disturbances and picky eating. We hypothesized that, compared with their typically developing peers, children with MID would show (i) less improvement in domains that generally decline with age (ADHD, oppositional, autistic, somatic, sleep, and picky eating) and (ii) greater increases in domains that typically rise (affective and anxiety problems). (iii) Differences in these associations were further explored across the three MID definitions.

## Methods

### Setting and participants

This study was part of the Generation R Study, a large, population-based cohort from Rotterdam, the Netherlands, designed to follow children’s development from foetal life to adolescence. Details can be found elsewhere [19]. The Medical Ethics Committee of Erasmus Medical Centre, Rotterdam, approved the study. Written informed assent/consent was obtained from all participants. For the present analyses, the final analytic sample consisted of children who completed both cognitive assessments and at least one parent-reported behavioural evaluation (*N* = 4,643) (Figure S1).

### Measures

#### Cognitive functioning

Cognitive functioning at age 13 was assessed using a short version of the Wechsler Intelligence Scale for Children, Fifth Edition (WISC-V) [20]. The WISC-V, widely used to evaluate children’s cognitive abilities, includes four subtests: Matrix Reasoning, Digit Span, Vocabulary, and Coding. These subtests were designed to assess specific cognitive domains and were used to estimate the Full Scale Intelligence Quotient (FSIQ). Trained research assistants conducted the assessments, with results recorded either on an iPad or through traditional paper methods.

#### Adaptive functioning

*Adaptive functioning* was assessed across three domains—conceptual, social, and practical functioning—based on DSM-5 criteria. Parent-reported items from various questionnaires across measurement waves were used to identify difficulties in these domains. These items were derived from multiple instruments, with partial overlap in measurement source with the outcome assessments. Additional details and a full list of items are provided in the Supplement.

*Conceptual Functioning* was assessed by determining whether the child attended a special education school, repeated grades, or had a parent-reported intellectual disability during assessments at ages 6, 10, and 14.

*Social Functioning* was measured using selected items from the Child Behavior Checklist (CBCL) at age 10 [21], the Social Responsiveness Scale (SRS) at age 6 [22], and the Behavior Rating Inventory of Executive Function (BRIEF) [23] at age 4. These items assessed the child’s capacity for social interactions, interpretation of social cues, and emotion regulation.

*Practical Functioning* was assessed using items that evaluated the child’s ability to manage daily tasks, such as hygiene, safety, and household chores. This domain was primarily operationalized using CBCL-derived items assessed at ages 1.5 and 10 years.

#### Psychopathology domains

Child mental health was assessed at five time points (1.5, 3, 5, 10, and 14 years) using age-appropriate Child Behavior Checklist (CBCL) versions [24]. We analysed eight DSM-oriented domains: affective, anxiety, ADHD, oppositional defiant, somatic, sleep, picky eating, and pervasive developmental problems, hereafter referred to as autism spectrum disorder (ASD) problems. Raw CBCL scores were converted to mean item scores and z-standardized across the analytic sample to allow comparison across age versions. Detailed item compositions are provided in the Supplement.

### Statistical analyses

All analyses were pre-registered (osf.io/k7562) and conducted using R version 4.3.2. Missing data on covariates were imputed using ‘*mice*’ package, which generated 25 imputed datasets [25]. Each imputed dataset was analysed separately, and the results were pooled into combined estimates using Rubin’s rules. A non-response analysis was conducted to assess potential selection bias due to missing WISC data. Participants included in the analytical sample were compared to those excluded on baseline characteristics (Table S1). To evaluate the construct validity of the adaptive functioning domains, we conducted confirmatory factor analyses (CFA) on the selected social and practical functioning items (Table S2-3). Detailed methods, factor loadings, and model fit indices are provided in the Supplement.

#### Operationalizing MID

The three operationalizations of MID were defined as follows:

1. IQ ≤ 75: Children were classified as having MID if they scored ≤ 75 on the WISC-V, without considering AF.
2. IQ ≤ 75 + AF: Children were classified as having MID if they scored ≤ 75 on the WISC-V and exhibited impairments in at least one of the following AF domains: conceptual, social, or practical functioning. Conceptual functioning impairments were defined as attending a special education school, following primary education in a special education setting, or repeating a grade at least once. Social functioning impairments were identified by scoring ≥ 2SD above the mean on the sum of social functioning items. Practical functioning impairments were identified by scoring ≥ 2SD above the mean on the sum of practical functioning items.
3. IQ ≤ 85 + AF: Children were classified as having MID if they scored ≤ 85 on the WISC-V and exhibited impairments in at least one of the AF domains (conceptual, social, or practical functioning) as described for MID2.

For each MID operationalization, participants with and without MID were matched on child age (at T1), sex, national origin, maternal educational attainment, and household income using Propensity Score Matching. Nearest neighbour matching was applied at a ratio of 1:3 (MID: control) using the R package ‘*MatchIt*’ [26] with default settings (no calliper, without replacement). Matching was conducted separately for each MID operationalization after multiple imputation. Balance after matching was evaluated using standardized mean differences, variance ratios, and Kolmogorov–Smirnov statistics, all indicating adequate balance (Supplementary Tables S5–S8, Figures S3–S6). Further details are provided in the Supplement.

#### Multilevel modelling

After matching, we employed linear mixed-effects models [27] to investigate the longitudinal trajectories of psychopathology from T1 to T5, stratified by MID status. Separate models were conducted for each DSM-oriented problem area (Depressive, Anxiety, ADHD, and ODD), while the trajectories of ASD problems, somatic problems, sleep problems, and picky eating symptoms were analysed across T1 to T3. Each linear mixed-effects model included fixed effects for age at assessment, MID status, and their interaction, enabling the examination of differences in psychopathology trajectories between children with and without MID over time. Age was modelled as a linear term to maintain parsimony and interpretability, given the limited number of measurement waves. Random intercepts and slopes were included to account for individual variability in baseline psychopathology levels and in the rate of change over time. Model convergence and random-effects variance estimates were inspected for all models to confirm adequate fit.

For each psychopathology domain, we specified the following fixed effects: intercept, age, MID status, and MID status*age. We report both the overall main effect (from models without the interaction) and the age interaction effect; where significant interactions were present, conditional main effects were additionally reported with age cantered at baseline.

Predicted trajectories of psychopathology domains for each operationalization were plotted to illustrate how these trajectories vary over time. To control for multiple comparisons across the primary outcomes (*n* = 8), a false discovery rate correction (FDR-Benjamini Hochberg) was applied [28] within each MID operationalization, in line with the preregistered analysis plan. MID4 was treated as an exploratory operationalization.

## Results

### Descriptive information

Table 1 summarizes the demographic characteristics of the MID and control groups across operationalizations. Male participants consistently constituted the majority in the MID groups, ranging from 64.2% to 69.6%. The percentage of participants with Dutch-origin mothers increased from 36.5% in MID1 to 52.5% in MID3, while the proportion in the lowest income group (< €1,200/month) decreased from 43.5% in MID1 to 27.7% in MID3. Similarly, the percentage of mothers with higher education levels increased from 22.6% in MID1 to 36.0% in MID3. These patterns suggest that broader definitions of MID (e.g., MID3) include individuals from more socioeconomically advantaged backgrounds compared to stricter definitions (e.g., MID1).

**Table 1:** Participant demographics per MID operationalization, and matched controls

	MID1 (IQ ≤ 75)		MID2 (IQ ≤ 75 + Conc., Soc., or Prac)		MID3 (IQ ≤ 85 + Conc., Soc., or Prac.)	
	MID	Control	MID	Control	MID	Control
N participants	115	345	82	246	408	1224
T1 age, mean (SD)	1.55 (0.10)	1.55 (0.08)	1.54 (0.08)	1.54 (0.10)	1.53 (0.08)	1.54 (0.10)
IQ, mean (SD)	69.1 (6.4)	100.0 (12.8)	68.3 (5.4)	99.7 (13.4)	75.2 (8.3)	102.0 (12.8)
IQ Range	45–74	76–140	45–74	76–139	45–84	76–139
Male, n (%)	80 (69.6)	240 (69.6)	57 (69.5)	171 (69.5)	262 (64.2)	790 (64.5)
Maternal national origin, n (%)						
Dutch	42 (36.5)	125 (36.2)	33 (40.2)	98 (39.8)	214 (52.5)	631 (51.6)
Non-Dutch European	9 (7.8)	27 (7.8)	6 (7.3)	18 (7.3)	25 (6.1)	71 (5.8)
Non-Dutch Non-European	64 (55.7)	193 (56)	43 (52.5)	130 (52.9)	169 (41.4)	522 (42.6)
Monthly household income (€/month), n (%)						
< 1,200	50 (43.5)	151 (43.8)	35 (42.7)	105 (42.7)	113 (27.7)	325 (26.6)
1,200-2,000	12 (10.4)	36 (10.4)	7 (8.5)	21 (8.5)	61 (15.0)	193 (15.8)
>2,000	53 (46.1)	158 (45.8)	40 (48.8)	120 (48.8)	234 (57.4)	706 (57.7)
Maternal education level, n (%)						
Primary or lower	26 (22.6)	76 (22.0)	17 (20.7)	50 (20.3)	55 (13.5)	163 (13.3)
Secondary	55 (47.8)	167 (48.4)	38 (46.3)	114 (46.3)	206 (50.5)	623 (50.9)
Higher	34 (29.6)	102 (29.6)	27 (32.9)	82 (33.3)	147 (36.0)	438 (35.8)

Note: Conc. = Conceptual functioning problems; Soc. = Social functioning problems; Prac. = Practical functioning problems; MID = Mild Intellectual Disability group; Control = Matched control for the MID group; IQ = Intelligence quotient based on the WISC-V

### Psychopathology domains (T1 to T5)

#### Affective problems

**Fig. 1:**
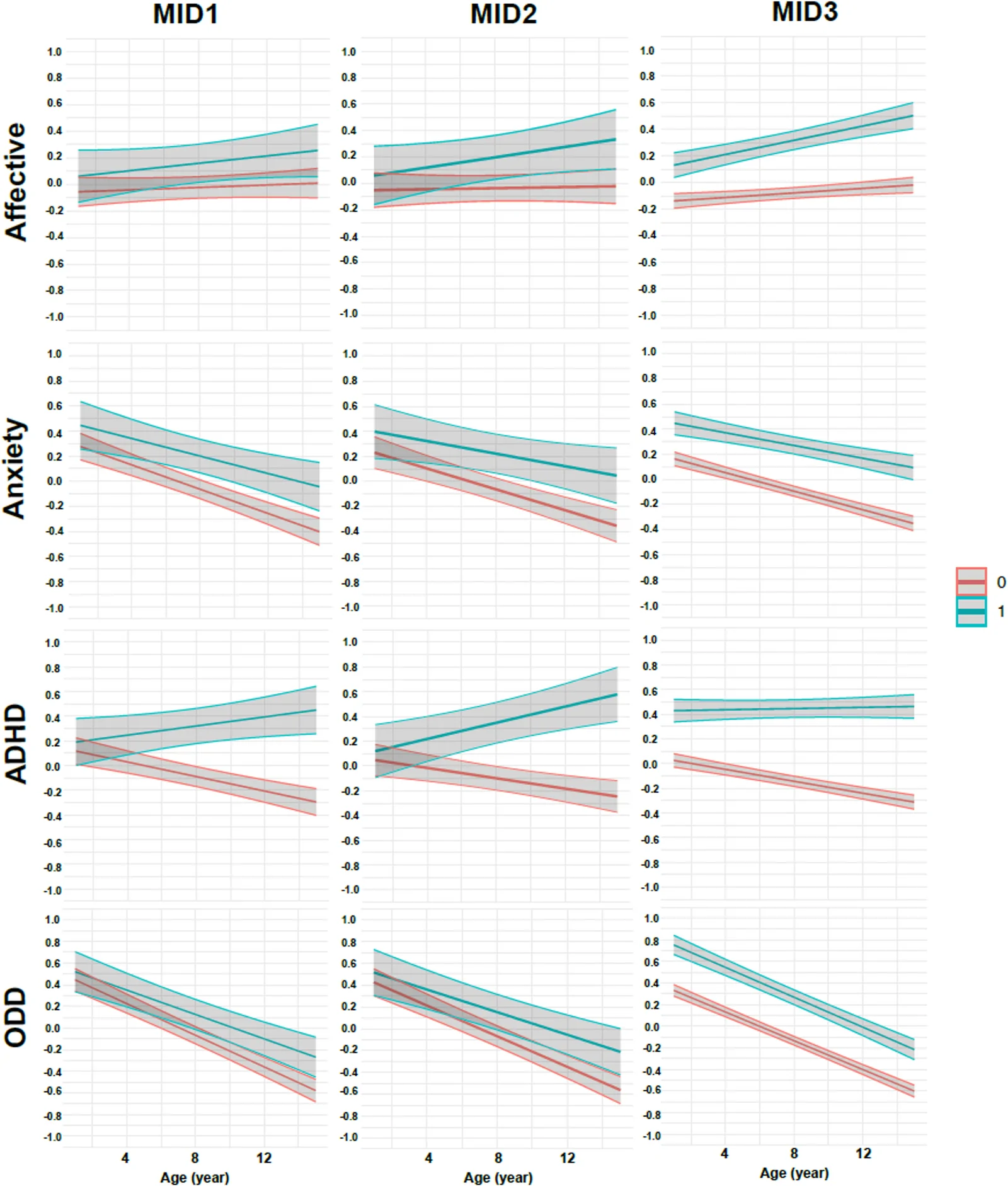
Longitudinal trajectories of psychopathology domains for children with MID and matched controls from T1 to T5. The figure presents longitudinal trajectories of psychopathology domains for children with MID (MID status = 1, represented in **blue**) and matched controls without MID (MID status = 0, represented in **red**) across three different MID operationalizations (MID1, MID2, and MID3). The shaded areas around the lines represent the confidence intervals for each trajectory. ADHD = Attention-deficit/hyperactivity problems; ODD = Oppositional defiant problems

Children with MID3 showed higher affective problems at baseline, and these differences further increased over time (β = 0.25, SE = 0.05, *p*FDR < 0.001; age interaction β = 0.01, SE = 0.00, *p*FDR = 0.004; Table 2; Fig. 1). MID1 and MID2 showed significantly higher affective problems compared to matched controls (MID1: β = 0.18, SE = 0.08, *p*FDR = 0.04; MID2: β = 0.23, SE = 0.09, *p*FDR = 0.02), but these differences did not change over time.

**Table 2:** Association between MID status and psychopathology domains over time

	MID1 (IQ ≤ 75)						MID2 (IQ ≤ 75 + Conc., Soc., or Prac.)						MID3 (IQ ≤ 85 + Conc., Soc., or Prac.)						
	Main effect			Age Interaction			Main effect			Age Interaction			Main effect				Age Interaction		
	Std β (SE)	*p*	pFDR	Std β (SE)	*p*	pFDR	Std β (SE)	*p*	pFDR	Std β (SE)	*p*	pFDR	Std β (SE)	*p*	pFDR	Std β (SE)		*p*	pFDR
Somatic Problems	0.03 (0.09)	0.72	0.80	-0.03 (0.03)	0.37	0.56	0.02 (0.11)	0.81	0.81	-0.05 (0.04)	0.14	0.21	0.25 (0.04)	< 0.001	**< 0.001**	-0.00 (0.01)		0.84	0.87
Picky eating	0.07 (0.09)	0.42	0.50	-0.04 (0.03)	0.19	0.35	0.12 (0.09)	0.23	0.26	-0.06 (0.03)	0.07	0.21	0.29 (0.04)	< 0.001	**< 0.001**	-0.01 (0.02)		0.73	0.79
ASD	0.26 (0.09)	0.008	0.01	0.06 (0.03)	0.06	0.18	0.31 (0.11)	0.005	0.01	0.05 (0.03)	0.17	0.21	0.47 (0.04)	< 0.001	**< 0.001**	0.08 (0.02)		< 0.001	**< 0.001**
Main effect at T1													0.25 (0.08)	0.001	**0.004**				
Sleep Problems	0.03 (0.09)	0.71	0.80	0.00 (0.03)	0.87	0.87	0.20 (0.07)	0.009	0.02	0.02 (0.03)	0.63	0.63	0.23 (0.04)	< 0.001	**< 0.001**	-0.00 (0.01)		0.65	0.75
Affective	0.18 (0.08)	0.03	**0.04**	0.01 (0.01)	0.40	0.56	0.23 (0.09)	0.01	0.02	0.02 (0.01)	0.16	0.21	0.38 (0.04)	< 0.001	**< 0.001**	0.01 (0.003)		0.001	**0.004**
Main effect at T1													0.25 (0.05)	< 0.001	**< 0.001**				
Anxiety	0.26 (0.07)	< 0.001	**0.001**	0.01 (0.01)	0.23	0.39	0.28 (0.07)	0.002	0.008	0.02 (0.01)	0.19	0.21	0.35 (0.04)	< 0.001	**< 0.001**	0.01 (0.01)		0.03	0.10
ADHD	0.40 (0.08)	< 0.001	**0.001**	0.04 (0.01)	< 0.001	.**001**	0.44 (0.09)	< 0.001	.**001**	0.05 (0.01)	< 0.001	.**001**	0.57 (0.04)	< 0.001	**< 0.001**	0.02 (0.002)		< 0.001	**< 0.001**
Main effect at T1	0.02 (0.11)	0.82	0.87				0.13 (0.08)	0.10	0.21				0.37 (0.05)	< 0.001	**< 0.001**				
ODD	0.19 (0.08)	0.02	.**03**	0.01 (0.01)	0.09	0.21	0.21 (0.09)	0.02	0.03	0.02 (0.01)	0.10	0.21	0.40 (0.04)	< 0.001	**< 0.001**	-0.00 (0.01)		0.61	0.75

Note. Linear mixed-effects models were used to test the associations between MID status and repeated psychopathology domains. Affective, anxiety, ADHD, and ODD problems were analysed from T1 to T5, whereas somatic problems, picky eating, ASD problems, and sleep problems were analysed from T1 to T3. Standardized effect estimates are shown for the main effect of MID status and the MID status × age interaction, together with standard errors (SE), p-values, and FDR-corrected p-values. Conc. = conceptual functioning problems; Soc. = social functioning problems; Prac. = practical functioning problems; MID = mild intellectual disability group; IQ = intelligence quotient based on the WISC-V; ASD = autism spectrum disorder problems; ADHD = attention-deficit/hyperactivity problems; ODD = oppositional defiant problems

#### Anxiety problems

Anxiety problems were significantly elevated across all MID groups (Table 2; Fig. 1). The largest main effect was observed for MID3 (β = 0.35, SE = 0.04, *p*FDR < 0.001), followed by MID2 (β = 0.28, SE = 0.07, *p*FDR = 0.008) and MID1 (β = 0.26, SE = 0.07, *p*FDR = 0.001). No significant age interactions were observed in any group.

#### ADHD problems

All MID groups showed significant age interactions, suggesting that ADHD problems increased over time compared to matched controls (Table 2; Fig. 1). MID3 showed significantly higher problems at baseline compared to controls (β = 0.37, SE = 0.05, *p*FDR < 0.001), with differences further widening over time (age interaction β = 0.02, SE = 0.00, *p*FDR < 0.001). In contrast, despite diverging trajectories over time, MID1 (β = 0.02, SE = 0.11, *p*FDR = 0.87) and MID2 (β = 0.13, SE = 0.08, *p*FDR = 0.21) showed no baseline differences.

#### ODD problems

Elevated ODD symptoms were observed in all MID groups (Table 2; Fig. 1), with the largest effect in MID3 (β = 0.40, SE = 0.04, *p*FDR < 0.001). Significant effects were also noted for MID1 and MID2, but no age interactions were found, indicating that differences in symptoms do not vary by age over time.

### Psychopathology domains (T1 to T3)

#### Somatic problems

**Fig. 2:**
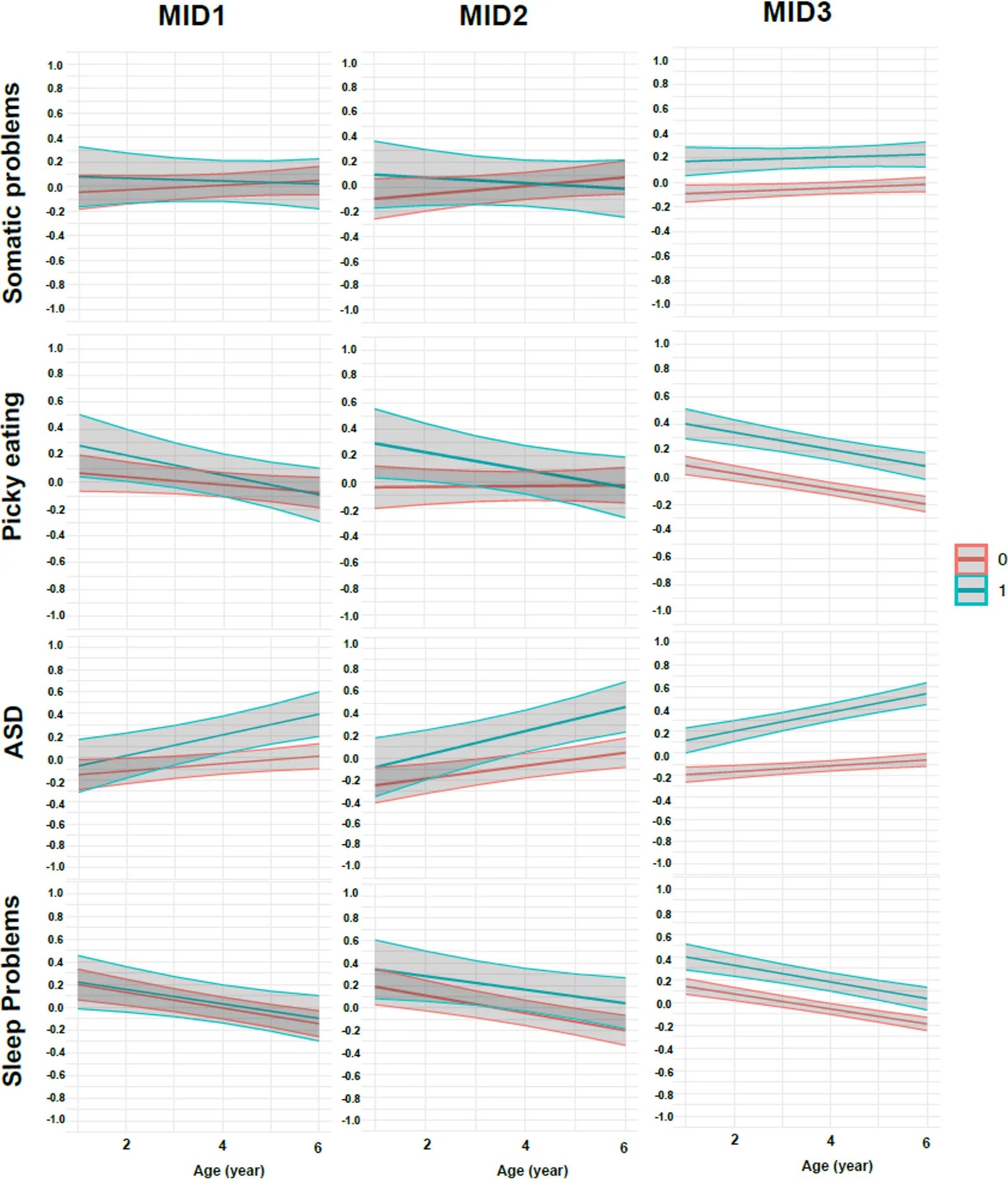
Longitudinal trajectories of psychopathology domains for children with MID and matched controls from T1 to T3. The figure presents longitudinal trajectories of psychopathology domains for children with MID (MID status = 1, represented in **blue**) and matched controls without MID (MID status = 0, represented in **red**) across three different MID operationalizations (MID1, MID2, and MID3). The shaded areas around the lines represent the confidence intervals for each trajectory. ASD = Autism Spectrum Disorder problems

No significant effects were observed for somatic problems in MID1 or MID2. However, MID3 demonstrated a significant main effect (β = 0.25, SE = 0.04, *p*FDR < 0.001), indicating higher levels of somatic problems in children with MID compared to controls, with no significant age interactions (Table 2; Fig. 2).

#### Picky eating problems

A significant main effect for picky eating problems was observed in MID3 (β = 0.29, SE = 0.04, *p*FDR < 0.001), with children with MID exhibiting higher levels of picky eating than controls. No significant age interactions were observed (Table 2; Fig. 2).

#### Autism Spectrum Disorder (ASD) problems

Children with MID3 showed significantly higher ASD problems at baseline (β = 0.25, SE = 0.08, *p*FDR = 0.004), and these differences increased further over time (age interaction β = 0.08, SE = 0.02, pFDR < 0.001; Table 2; Fig. 2). MID1 (β = 0.26, SE = 0.09, *p*FDR = 0.01) and MID2 (β = 0.31, SE = 0.11, *p*FDR = 0.01) also showed higher baseline symptoms, but no significant changes in trajectories.

#### Sleep problems

Significant main effects for sleep problems were observed in MID2 (β = 0.20, SE = 0.07, *p*FDR = 0.02) and MID3 (β = 0.23, SE = 0.04, *p*FDR < 0.001), indicating elevated sleep problems in children with MID compared to controls. No significant age interactions were observed in any group (Table 2; Fig. 2).

## Discussion

This longitudinal study examined the developmental trajectories of behavioural and emotional problems in children with MID compared to matched controls, using three MID definitions reflecting changes in DSM criteria and common practice. Our findings revealed that children with MID exhibit higher levels of behavioural and emotional problems. The findings across operationalizations for ADHD, ODD, affective, anxiety, and ASD problems among children with MID align with prior research highlighting the increased vulnerability of children with mild intellectual or developmental impairments [3–7, 29], and we showed that this vulnerability is persistent in the age ranges studied. In addition, somatic problems (MID3), picky eating (MID3), and sleep problems (MID2 and 3) were higher in MID than non-MID participants, indicating persistent vulnerability.

Moreover, the higher vulnerability of children with MID compared to non-MID peers increased over time for ADHD (all operationalizations), and for affective (MID3) and ASD problems (MID3). This did not hold for the other psychopathology domains, so findings partially support our hypothesis. We hypothesized that problems that tend to decrease over time in the general population would decrease less in children with MID, while problems that tend to increase would increase more in children with MID. In line with this, across MID operationalizations, ADHD symptoms remained either stable or increased over time in children with MID, while non-MID participants showed decreasing ADHD symptom trajectories, suggesting that children with MID may face prolonged difficulties with ADHD symptoms [6, 7]. Additionally, affective and ASD problems in MID3 (but not MID1 and 2) showed a significantly greater increase over time compared to matched controls. Of note, we hypothesized a smaller *decrease* in ASD symptoms in children with MID, but found an *increase* over time relative to children without MID who, indeed, decreased. Because parts of the adaptive functioning construct were derived from CBCL-based items, and some of these items overlap directly or conceptually with the outcome measures, shared measurement content may have contributed to the observed associations. Therefore, these findings should be interpreted with caution. Problems in AF may overlap with mental health problems, and adding AF to the operationalization in MID1 and MID2 may therefore increase mental health problems. Focusing further on the results according to differences in MID operationalizations, we found that compared to MID1 (IQ ≤ 75), adding AF criteria (MID2) resulted in only slightly stronger associations across most psychopathology domains, except for a notable effect on sleep problems. In contrast, further expanding the IQ threshold from ≤ 75 to ≤ 85 and incorporating adaptive impairments (MID3) revealed more pronounced behavioural and emotional problems compared to MID1 and 2. Together, the findings suggest that (a) a MID operationalization based solely on IQ (IQ ≤ 75) already identifies children with elevated behavioural and emotional problems and (b) the higher level of problems found in the MID3 compared to the MID2 operationalization, which apply the same AF criteria, seems to come from increasing the IQ threshold (from ≤ 75 in MID2 to ≤ 85 in MID3), although some contribution of shared measurement content cannot be fully excluded. The results for the MID3 group are consistent with previous findings, indicating that individuals with IQs in the borderline range (75–85) experience significant psychosocial and mental health needs [30–32]. In fact, our results suggest that they have more mental health needs than the subset of children with lower IQ (i.e., MID1 and 2).

A strength of this study is its comprehensive examination of various MID operationalizations within a large dataset. To our knowledge, the current study and the study by are the first studies to test psychopathology trajectories across multiple commonly used MID definitions, providing valuable insights into how different diagnostic criteria capture distinct profiles of behavioural and emotional problems in children. In doing so, the findings raise important questions about the conceptual boundaries of MID. Expanding diagnostic criteria to include a broader range of cognitive and AF difficulties may lead to increasing overlap with mental health problems. This raises concerns about whether MID is evolving into an overly broad category that encompasses a very diverse set of mental health, AF, and cognitive difficulties rather than a clearly defined diagnostic group. While our study cannot provide definitive answers to this, it highlights the need for continued reflection on how to best delineate MID from mental health problems. Alternatively, reflection may also lead to the conclusion that this differentiation is irrelevant and that a broad MID group is not overly broad as long as those in need receive appropriate support. A second strength of this study is that it contributes to the sparse literature on sleep problems and picky eating in children with MID [3]. Higher levels of sleep problems in MID2 and MID3 may indicate that AF problems may exacerbate sleep problems, which, in turn, could impair daily functioning and overall quality of life [33, 34]. Picky eating was also significantly elevated in MID3, drawing attention to an area that may be related to nutritional status and growth. These findings suggest the need for comprehensive assessments that encompass behavioural and emotional as well as functional areas, enabling more targeted support for children with MID and their parents [35, 36].

Several methodological limitations warrant consideration. First, some criteria used to define AF overlapped with items included in the outcome measures of behavioural and emotional problems. This overlap may have influenced the observed associations and could partially explain the relationships identified in the study. However, the absence of clear differences between MID1 and MID2 argues against this explanation as the sole driver of the findings. Second, while we controlled for key demographics (age, sex, national origin, maternal education, and household income) using propensity score matching, unmeasured factors -including parenting styles, environmental stressors, and genetic predispositions- could have influenced the findings. We did not include these variables in the matching, as they may act as mediators or colliders rather than confounders, and therefore do not invalidate the current findings. Third, reliance on parent-reported measures for AF and behavioural/emotional problems, rather than a clinician, may introduce biases. Although AF and mental health problems conceptually overlap, a clinician may still be better able to differentiate between them to the extent that differentiation is possible. In addition, although confirmatory factor analyses supported the intended AF domain structure, the high correlation between domains indicates substantial overlap between constructs. Furthermore, standardized adaptive functioning instruments such as the Vineland Adaptive Behavior Scales [37] were not available in this cohort, limiting the ability to directly assess the convergent validity of the constructed AF domains. Fourth, and related to this, none of the MID definitions used in this study incorporated clinical judgment, even though DSM-5 explicitly states that an MID diagnosis should not rely solely on test scores. The absence of qualitative, clinician-driven assessments may mean that some children who would be diagnosed in clinical settings were not identified as MID in this study, and vice versa. In addition, detailed information on genetic aetiology and formal neurodevelopmental or psychiatric comorbidities was not consistently available across the cohort, precluding stratified analyses and adjustment for comorbid conditions. Therefore, the findings likely reflect the overall symptom burden associated with MID. Fifth, group sizes and, therefore, power, differed between MID operationalizations, which is why MID group comparisons were also based on effect sizes. However, estimation accuracy differed among the three MID groups, although the smallest MID2 group was still substantial (*n* = 82). Finally, exclusion of participants without available WISC data may have introduced selection bias, which could limit the generalizability of the findings.

Our study underscores that both narrower (IQ ≤ 75) and particularly broader (IQ ≤ 85) definitions of MID capture children at risk of persistent behavioural and emotional problems. In addition, mostly for the latter group, the increasing risk of psychopathology from childhood into adolescence for ASD, affective, and ADHD problems should be interpreted with caution, as data on perceived or provided support were not available, and causal mechanisms cannot be determined. That is, the presence of mental health problems on top of MID may further hamper adaptive and daily life functioning. Children with an IQ score between 75 and 85 may have less access to specialized care and facilities than children with lower intellectual functioning.

Our results also have implications for research. Various MID operationalizations may result in different associations with external variables, possibly affecting the implications of study results. Not including AF in MID operationalizations, as has been common in research for practical reasons [38], may lead to underestimation of associations with psychopathology problems when a DSM-5-TR definition of MID is accepted. MID operationalizations should therefore be taken into account when comparing study results, and MID definitions should be harmonized in future research.

In conclusion, this study showed that children with MID, particularly when defined as IQ ≤ 85 and including AF impairments, exhibit persistent as well as increasing behavioural and emotional problems compared to their matched peers. Standardizing MID criteria in research and clinical practice is needed to improve comparability between studies and enhance diagnosis accuracy.

## Supplementary Information

Below is the link to the electronic supplementary material.

Supplementary Material 1 (DOCX 376 KB)

## Data Availability

Data can be obtained upon request. Requests should be directed towards the management team of the Generation R Study (secretariaat.genr@erasmusmc.nl), which has a protocol for approving data requests. Because of restrictions based on privacy regulations and informed consent of participants, data cannot be made freely available in a public repository.
