# Supplementary material for: Psychopathology from childhood to adolescence in children with and without Mild Intellectual Disability (MID): a comparison across three MID operationalizations
Source: Eur Child Adolesc Psychiatry. 2026 Jun 22;35(10):3335–45. doi: 10.1007/s00787-026-03106-3 (PMC13701136; doi:10.1007/s00787-026-03106-3)
Supplement: Supplementary file 1 [file 787_2026_3106_MOESM1_ESM.docx]

**Supplementary Information**

**Psychopathology from Childhood to Adolescence in children with and without Mild Intellectual Disability (MID): a comparison across three MID operationalizations**

Dogukan Koc^1,2^, Maurits Masselink^3,4,5^, Stefania Barzeva^3,5^, Daan H. G. Hulsmans^3,6^, Nanda N. J. Rommelse^7,8^, Roy Otten^3^, Catharina A Hartman^5^, Pauline W. Jansen*^1,2,9^

^1^Department of Child and Adolescent Psychiatry/Psychology, Erasmus MC, University Medical Centre Rotterdam – Sophia, Rotterdam, the Netherlands

^2^Generation R Study Group, Erasmus University Medical Centre, Erasmus University Rotterdam, Rotterdam, the Netherlands

^3^ Behavioral Science Institute, Radboud University, Nijmegen, The Netherlands

^4^ Department of Psychiatry, Radboud University Medical Center, Nijmegen, The Netherlands

^5^ Department of Psychiatry, University Medical Center Groningen, Groningen, The Netherlands

^6^ Faculty of Behavioural and Social Sciences, University of Groningen, Groningen, The Netherlands

^7^ Karakter Child and Adolescent Psychiatry University Centre, The Netherlands

^8^ Developmental Psychology, Utrecht University, Utrecht, the Netherlands

^9^ Department of Psychology, Education and Child Studies, Erasmus School of Social and Behavioral Sciences, Erasmus University Rotterdam, the Netherlands

*Corresponding author, email: **p.w.jansen@erasmusmc.nl**

**Supplementary Methods**

**Measures**

**Conceptual Functioning Assessment:**

Conceptual functioning assessment involves evaluating the capacity to independently learn and utilize new knowledge, skills, and competencies. This evaluation is derived from specific items reported by parents within the Generation R cohort, focusing on three main indicators:

1. **Attends Special Education School:**

This is identified through parent responses indicating whether the child attends a special education institution.

2. **Special Education Primary School:**

Identification of special education attendance at specific ages (at age 6 years: "special education at age 6 yrs.", at age 10 years: "Does the child receive special education, remedial services, or attend a special class or special school?").

3. **Repeating Grade:**

Information was captured in response to questions, with a note that it considers "repeating grade 1 time."

**Social** **Functioning Assessment:**

Social functioning assessment within the Generation R study measures an individual's capacity to navigate and adhere to societal norms, comprehend social cues and contexts, and manage emotional responses. It is based on eight items sourced from various parent-reported questionnaires, including the Child Behavior Checklist (CBCL) [1], the Social Responsiveness Scale (SRS) [2], and the Behavior Rating Inventory of Executive Function (BRIEF) [3]. These items were selected according to three criteria inspired by the DSM-5's guidelines for assessing adaptive functioning in the social domain:

(1) The item reflects social functioning, i.e. pertaining to social interactions, friendships, social cues, communication, conversation, language, emotion regulation, understanding of risk in social situations, and risk for manipulation by others.
(2) The item reflects typical or normal behavior, one that is common or present across any developmental age.
(3) The item reflects immaturity or a delay in an age-relevant skill or behavior. Items that are not selected should reflect attitudes, habits, or behaviours consistent with children < 11 years old.

For an item to be included in the social functioning construct, all criteria had to be met. Any discrepancies between the raters were discussed and resolved. The final selection of social functioning items can be found in Table S2. Items were rated on a 3-point scale (1 = *Never*; 2 = *Sometimes*; 3 = *Very often*). See also the footnote in Table S2 explaining how items from different measures were brought to the same scale.

**Practical** **Functioning Assessment:**

Practical functioning assessment measures an individual's capability to independently manage personal and day-to-day tasks, aligning with age-appropriate levels of self-care and safety. This assessment incorporates ten items from the CBCL, evaluated against three criteria derived from the DSM-5 descriptions of adaptive functioning in the practical domain:

(1) The item reflects practical functioning, i.e., pertaining to personal care around health and safety, meals, dressing, elimination, hygiene; daily living tasks, household chores, and recreational skills.
(2) The item reflects typical or normal behaviour, one that is common or present across any developmental age.
(3) The item reflects immaturity or a delay in an age-relevant skill or behaviour. Items that are not selected should reflect attitudes, habits, or behaviours consistent with children < 11 years old.

For an item to be included in the practical functioning construct, all three criteria had to be met. Any discrepancies between the raters were discussed and resolved. The final selection of social functioning items can be found in Table S3. Items rated on a 3-point scale (1 = *Never*; 2 = *Sometimes*; 3 = *Very often*). See also the footnote in Table S3 explaining how items from different measures were brought to the same scale.

**Confirmatory Factor Analysis of Adaptive Functioning Domains**

To evaluate whether the selected items adequately reflected the intended adaptive functioning domains, we conducted confirmatory factor analyses (CFA) for the social and practical functioning constructs.

The CFA models were specified using a two-factor structure (social and practical functioning), in which each item loaded on its respective latent factor. Because items were derived from different instruments and measurement waves, we allowed for correlated residuals between items originating from the same assessment wave and between conceptually overlapping items. This approach was chosen to account for shared method variance and developmental clustering.

Items were treated as continuous variables, consistent with their summed score construction and subsequent z-standardization. Models were estimated using maximum likelihood estimation.

Model fit was evaluated using standard indices, including the Comparative Fit Index (CFI), Tucker–Lewis Index (TLI), Root Mean Square Error of Approximation (RMSEA), and Standardized Root Mean Square Residual (SRMR). Models were specified and evaluated following standard recommendations [4] and were estimated using the lavaan package in R [5]

**Psychopathology Domains**

The CBCL was used to assess child mental health problems at various time points. At T1 (1½ years), T2 (3 years), and T3 (5 years), the CBCL version for ages 1.5–5 years, consisting of 99 items, was administered. At T4 (10 years), and T5 (14 years), the CBCL version for ages 6–18 years, with 112 items, was used. Primary caregivers completed these questionnaires, which generated DSM-oriented problem scales for both CBCL versions, including affective problems, anxiety problems, ADHD, and oppositional defiant disorder (ODD) [6]. We additionally included the pervasive developmental, somatic, sleep, and picky eating problems as assessed in the CBCL version 1.5–5 at T1, T2, and T3 [6]. A sleep problems score was calculated at each time point (T1 to T3) by summing five dyssomnia items: ‘Doesn't want to sleep alone’; ‘Has trouble getting to sleep’; ‘Resists going to bed at night’; ‘Sleeps less than most kids during day and/or night’; and ‘Wakes up often at night’. This selection of items was based on a factor analysis conducted in the Generation R cohort, as described by Kocevska et al. (2017) [7]. A picky eating symptoms score was calculated at each time point (T1 to T3) by summing two items: (i) ‘does not eat well’ and (ii) ‘refuses to eat’, following a previous approach [8]. CBCL raw scores were converted to mean item scores for comparability across versions (1.5–5 and 6–18 years) and subsequently z-standardized across the analytic sample, yielding estimates in standard deviation units.

**Covariates**

Maternal covariates included age at enrolment in the study, educational attainment, and household income [9]. Educational attainment was categorized as 3 years of secondary school or less (typically corresponds with 11 years of education); more than 3 years of secondary school or intermediate vocational training (generally corresponds with 12–15 years of education); higher vocational training (typically corresponds to 16 or 17 years of education), and university degree (usually indicates 18 years of education or more). Parents self-reported their household income during pregnancy, and it was categorized into less than €1200,- (below the poverty level), €1200,- to €2000,-(low income in 2005), and more than €2000,-per month. The covariates of the child were sex and age at assessments and national origin. The child's national origin was categorized as Dutch, Western, and non-Western, with a non-Dutch origin being assigned if one of the parents was born abroad. The Western category included those with a European or North American origin. Those with a non-Western origin included Surinamese, Dutch Antillean, Turkish, African, and Asian descent.

**Participant Matching**

Propensity Score Matching (PSM) is a method used to balance two or more groups on confounding factors, enabling observational data to be comparable when testing for group differences on one or more outcomes. The matching procedure was intended to estimate the average treatment effect for the treated (ATT), comparing children with MID to matched non-MID controls with similar observed background characteristics. Participants classified under any of the three MID operationalizations were excluded from serving as matched controls in any other operationalization. The following modelling procedures were repeated for each MID operationalization. For descriptive purposes, means of continuous variables and proportions (%) of categorical variables are presented for each MID operationalization and their matched controls.

**Deviations from pre-registration**

During the course of the project, we revised the definition of MID2. In the preregistration, MID2 was defined as IQ ≤ 85 in combination with deficits in the conceptual domain. For the current analyses, MID2 was redefined as IQ ≤ 75 in combination with deficits in at least one domain (conceptual, social, or practical), to better align with DSM-5 criteria. The preregistered definition (IQ ≤ 85 with conceptual deficits) was retained as an additional exploratory operationalization, hereafter referred to as MID4. Results for MID4 are presented in Table S11.

**Supplementary Results**

**Confirmatory Factor Analysis**

The CFA showed that most items had significant loadings on their respective latent factor (p < .001), with moderate to strong standardized loadings observed across social and practical domains (Table S4), with good a good model fit (CFI = 0.996, TLI = 0.995, RMSEA = 0.031 [90% CI: 0.028–0.034], SRMR = 0.072).

One practical functioning item (prac3; bedwetting) showed a low and non-significant loading, indicating limited contribution to the latent factor in this sample. However, because the CFA aimed to assess the fit of the predefined adaptive functioning structure rather than to refine the scale post hoc, the item was retained on conceptual grounds.

The correlation between the latent social and practical functioning factors was high (r = .96, p < .001), indicating near-perfect overlap between domains. Strong overlap between AF domains has been found by others as well, and there is an ongoing debate about uni-versus multidimensionality of AF [3–5]. Although we cannot exclude the possibility that the domain overlap in the current study is the result of our specific AF operationalization, we consider it likely that the overlap largely reflects the broader dimensionality question of AF.

**MID Operationalization and Matching**

The operationalization of MID identified varying proportions of participants across the study population (Table S9). MID1 included 115 participants (2.5%), MID2 included 82 participants (1.8%), and MID3 comprised 408 participants (8.8%). Overall, 441 children (9.5%) met the criteria under at least one definition. Figure S2 illustrates the overlap among operationalizations, with 82 participants identified across all three definitions, 33 unique to MID1 and 326 unique to MID3. These overlaps highlight differences between IQ-based definitions (e.g., MID1) and those incorporating adaptive functioning (e.g., MID3). Propensity score matching ensured comparability between MID and non-MID groups. Post-matching, all standardized mean differences (SMDs) were below or near the threshold of 0.1, variance ratios were close to 1, and Kolmogorov-Smirnov statistics indicated minimal differences in variable distributions. These results indicate a good balance between groups (Tables S5–S8, Figures S3–S6). To aid interpretation of the MID operationalizations, we examined the distribution of adaptive functioning (AF) indicators across groups (Table S10). The proportion of children with conceptual impairment increased from 69.6% in MID1 to 97.6% in MID2 and to 100% in MID4, while only 57.3% of children in MID3 showed conceptual impairment, reflecting the inclusion of individuals with social or practical difficulties. Social and practical functioning impairments were most pronounced in MID3, whereas MID1, MID2, and MID4 showed more comparable distributions.

*N = 9901*

*A child with WISC assessment*

*n = 4863*

*A child with WISC and at least one CBCL 1.5-6 assessment*

*n = 4643*

5038 with no WISC assessment

220 with no CBCL data T1 to T3 and missing data on SRS and BRIEF questioners

**Figure S1.** Flowchart of the study participants.

**Figure S2.** MID status overlaps across operationalization groups.

**Figure S3.** Absolute standardized mean differences and Kolmogorov-Smirnov statistics between MID and matched controls, shown before (represented by red circles labelled 'unmatched') and after (represented by blue triangles labelled 'matched') the matching process in MID operationalization 1.

**Figure S4.** Absolute standardized mean differences and Kolmogorov-Smirnov statistics between MID and matched controls, shown before (represented by red circles labelled 'unmatched') and after (represented by blue triangles labelled 'matched') the matching process in MID operationalization 2.

**Figure S5.** Absolute standardized mean differences and Kolmogorov-Smirnov statistics between MID and matched controls, shown before (represented by red circles labelled 'unmatched') and after (represented by blue triangles labelled 'matched') the matching process in MID operationalization 3.

**Figure S6.** Absolute standardized mean differences and Kolmogorov-Smirnov statistics between MID and matched controls, shown before (represented by red circles labelled 'unmatched') and after (represented by blue triangles labelled 'matched') the matching process in MID operationalization 4.

| **Table S1.** Comparison of participants included vs not included in MID analyses | | | |
| --- | --- | --- | --- |
| **Characteristic** | **Not included (N = 5,259)** | **Included (N = 4,643)** | **p-value** |
| **Gender** |  |  | 0.003 |
| Male | 2,738 (52%) | 2,277 (49%) |  |
| Female | 2,520 (48%) | 2,366 (51%) |  |
| **Maternal ethnicity** |  |  | <0.001 |
| Dutch | 2,165 (41%) | 2,784 (60%) |  |
| Non-Dutch European | 457 (8.7%) | 397 (8.6%) |  |
| Non-Dutch Non-European |  |  |  |
| Caribbean | 778 (15%) | 440 (9.5%) |  |
| Moroccan/Turkish | 1,090 (21%) | 453 (9.8%) |  |
| African | 457 (8.7%) | 300 (6.5%) |  |
| Indonesian | 311 (5.9%) | 269 (5.8%) |  |
| **Maternal education** |  |  | <0.001 |
| Primary or lower maternal education | 829 (16%) | 323 (7.0%) |  |
| Secondary maternal education | 2,707 (51%) | 1,887 (41%) |  |
| Higher maternal education | 1,722 (33%) | 2,433 (52%) |  |
| **Household income** |  |  | <0.001 |
| Low income (< 1200 €/month) | 1,729 (33%) | 757 (16%) |  |
| Middle (1200-2000 €/month) | 926 (18%) | 633 (14%) |  |
| Higher (>2000 €/month) | 2,603 (50%) | 3,253 (70%) |  |
| *Note:* Values are presented as n (%). Differences between included and excluded participants were assessed using Pearson’s χ² tests for categorical variables. | | | |

| **Table S2.** Social functioning items. | | | | |
| --- | --- | --- | --- | --- |
| **Item (NL)** | **Item (EN)** | **Questionnaire** | **Item code** | |
| Doet te jong voor zijn/haar leeftijd? | Acts too young for his/her age | CBCL/6-18 (or 1.5-5 if 6-18 is missing) | soc1 |  |
| Gaat liever om met jongere jongens of meisjes. | Prefers to be with younger boys/girls | CBCL/6-18 | soc2 |  |
| Speelt met eigen geslachtsdelen in het openbaar. | Plays with own genitals in public. | CBCL/6-18 (or 1.5-5 if 6-18 is missing) | soc3 |  |
| Stopt niet met lachen om grappige dingen als anderen dat doen. | Doesn't stop laughing at funny things when others do. | BRIEF 4 | soc4 |  |
| Is zich er niet van bewust wanneer zijn/haar gedrag negatieve reacties veroorzaakt | Is not aware when his/her behavior causes negative reactions. | BRIEF 4 | soc5 |  |
| is helemaal niet in staat om de eigenlijke betekenis van gesprekken met oudere kinderen of volwassenen te snappen. | is completely unable to understand the actual meaning of conversations with older children or adults. | SRS 6 | soc6 |  |
| heeft moeite om het verloop van een gewoon gesprek met andere kinderen te volgen. | has difficulty following the flow of an ordinary conversation with other children. | SRS 6 | soc7 |  |
| geeft een ongewone of onlogische reden voor wat hij/zij doet. | gives an unusual or illogical reason for what he/she is doing. | SRS 6 | soc8 |  |
| *Note:* For the Social Functioning score, items from both CBCL/1.5-5 and CBCL/6-18 age groups were included. Items from T1 and T4 assessments were utilized. In cases of missing data for either T1 or T4, items from the first available measurement wave were used. Scores from the SRS (4-point scale) were rescaled by multiplying by 0.75 to align with the 3-point scale of the CBCL and BRIEF. The rescaled scores for each individual across the selected items were summed up to obtain the Social Functioning score. Z-scores were calculated for each individual. | | | | |

| **Table S3.** Practical functioning items. | | | |
| --- | --- | --- | --- |
| **Item (NL)** | **Item (EN)** | **Questionnaire** | **Item code** |
| Doet ontlasting (poept) buiten de wc of in de broek | Defecates (poops) outside the toilet or in the pants | CBCL/6-18 | prac1 |
| Broekplassen overdag | Pants peeing during the day | CBCL/6-18 | prac2 |
| Bedplassen | Bedwetting | CBCL/6-18 | prac3 |
| Klampt zich vast aan volwassenen of is te afhankelijk | Clings to adults or is too dependent | CBCL/6-18 (or 1.5-5 if 6-18 is missing) | prac4 |
| Kauwt op dingen die niet eetbaar zijn | Chews on things that are inedible | CBCL/1-5.5 | prac5 |
| Wil voortdurend geholpen worden | Wants constant help | CBCL/1-5.5 | prac6 |
| Eet of drinkt dingen die eigenlijk niet eetbaar of drinkbaar zijn (snoep niet meetellen) (schrijf op): | Eats or drinks things that are not actually edible or drinkable (not counting sweets) (write down): | CBCL/1-5.5 | prac7 |
| Loopt zomaar weg | Just walks away | CBCL/1-5.5 | prac8 |
| Onhandig of stuntelig. | Clumsy | CBCL/6-18 | prac9 |
| Smeert of speelt met ontlasting | Smears or plays with defecates | CBCL/1-5.5 | prac10 |
| *Note:* For the Practical Functioning score, items from both CBCL/1.5-5 and CBCL/6-18 age groups were included. Items from T1 and T4 assessments were utilized. In cases of missing data for either T1 or T4, items from the first available measurement wave were used. The scores for each individual across the selected items were summed up to obtain the Practical Functioning score. Z-scores were calculated for each individual. | | | |

| **Table S4.** Standardized factor loadings and factor correlation from confirmatory factor analysis | | | |
| --- | --- | --- | --- |
| **Factor** | **Item** | **Standardized loading** | **p-value** |
| Social | soc1 | 0.648 | <.001 |
| Social | soc2 | 0.559 | <.001 |
| Social | soc3 | 0.498 | <.001 |
| Social | soc4 | 0.338 | <.001 |
| Social | soc5 | 0.416 | <.001 |
| Social | soc6 | 0.406 | <.001 |
| Social | soc7 | 0.663 | <.001 |
| Social | soc8 | 0.592 | <.001 |
| Practical | prac1 | 0.286 | <.001 |
| Practical | prac2 | 0.234 | .004 |
| Practical | prac3 | 0.059 | .233 |
| Practical | prac4 | 0.535 | <.001 |
| Practical | prac5 | 0.297 | <.001 |
| Practical | prac6 | 0.313 | <.001 |
| Practical | prac7 | 0.265 | <.001 |
| Practical | prac8 | 0.313 | <.001 |
| Practical | prac9 | 0.457 | <.001 |
| Practical | prac10 | 0.447 | <.001 |
| *Note:* The correlation between the latent Social and Practical factors was high (r = .958, p < .001). Model fit indices indicated good model fit (CFI = 0.996, TLI = 0.995, RMSEA = 0.031, 90% CI = 0.028–0.034, SRMR = 0.072). CFI = Comparative Fit Index; TLI = Tucker–Lewis Index; RMSEA = Root Mean Square Error of Approximation; CI = confidence interval; SRMR = Standardized Root Mean Square Residual. | | | |

| **Table S5.** Propensity score matching results for MID1 | | | | | | |
| --- | --- | --- | --- | --- | --- | --- |
|  | **Means Treated** | **Means Control** | **Std. Mean Diff.** | **Var. Ratio** | **eCDF Mean** | **eCDF Max** |
| Distance | 0.0413 | 0.0413 | -0.0009 | 1.003 | 0.0006 | 0.0145 |
| Male | 0.6667 | 0.7101 | -0.0922 | . | 0.0435 | 0.0435 |
| Female | 0.3333 | 0.2899 | 0.0922 | . | 0.0435 | 0.0435 |
| Dutch | 0.4493 | 0.4879 | -0.0777 | . | 0.0386 | 0.0386 |
| Non-Dutch European | 0.0725 | 0.0918 | -0.0745 | . | 0.0193 | 0.0193 |
| Non-European  Caribbean | 0.1449 | 0.1159 | 0.0823 | . | 0.029 | 0.029 |
| Non-European  Moroccan/Turkish | 0.1884 | 0.1836 | 0.0124 | . | 0.0048 | 0.0048 |
| Non-European  African | 0.1159 | 0.1063 | 0.0302 | . | 0.0097 | 0.0097 |
| Non-European  Indonesian | 0.029 | 0.0145 | 0.0864 | . | 0.0145 | 0.0145 |
| Primary or lower maternal education | 0.1159 | 0.1739 | -0.1811 | . | 0.058 | 0.058 |
| Secondary maternal education | 0.5217 | 0.4638 | 0.1161 | . | 0.058 | 0.058 |
| Higher maternal education | 0.3623 | 0.3623 | 0 | . | 0 | 0 |
| Low income (< 1200 €/month) | 0.3188 | 0.3188 | 0 | . | 0 | 0 |
| Middle (1200-2000 €/month) | 0.1014 | 0.1063 | -0.016 | . | 0.0048 | 0.0048 |
| Higher (>2000 €/month) | 0.5797 | 0.5749 | 0.0098 | . | 0.0048 | 0.0048 |
| Age at T1 | 1.5549 | 1.5412 | 0.1402 | 1.3402 | 0.0187 | 0.0773 |

| **Table S6.** Propensity score matching results for MID2 | | | | | | |
| --- | --- | --- | --- | --- | --- | --- |
|  | **Means Treated** | **Means Control** | **Std. Mean Diff.** | **Var. Ratio** | **eCDF Mean** | **eCDF Max** |
| Distance | 0.0639 | 0.0638 | 0.0012 | 1.0103 | 0.0006 | 0.0125 |
| Male | 0.6391 | 0.6366 | 0.0052 |  | 0.0025 | 0.0025 |
| Female | 0.3609 | 0.3634 | -0.0052 |  | 0.0025 | 0.0025 |
| Dutch | 0.5414 | 0.5689 | -0.0553 |  | 0.0276 | 0.0276 |
| Non-Dutch European | 0.0451 | 0.0351 | 0.0483 |  | 0.01 | 0.01 |
| Non-European  Caribbean | 0.1429 | 0.1504 | -0.0215 |  | 0.0075 | 0.0075 |
| Non-European  Moroccan/Turkish | 0.1729 | 0.1454 | 0.0729 |  | 0.0276 | 0.0276 |
| Non-European  African | 0.0451 | 0.0551 | -0.0483 |  | 0.01 | 0.01 |
| Non-European  Indonesian | 0.0526 | 0.0451 | 0.0337 |  | 0.0075 | 0.0075 |
| Primary or lower maternal education | 0.0902 | 0.0827 | 0.0262 |  | 0.0075 | 0.0075 |
| Secondary maternal education | 0.5489 | 0.5815 | -0.0655 |  | 0.0326 | 0.0326 |
| Higher maternal education | 0.3609 | 0.3358 | 0.0522 |  | 0.0251 | 0.0251 |
| Low income (< 1200 €/month) | 0.2406 | 0.2531 | -0.0293 |  | 0.0125 | 0.0125 |
| Middle (1200-2000 €/month) | 0.1353 | 0.1404 | -0.0147 |  | 0.005 | 0.005 |
| Higher (>2000 €/month) | 0.6241 | 0.6065 | 0.0362 |  | 0.0175 | 0.0175 |
| Age at T1 | 1.5327 | 1.5397 | -0.1033 | 0.6224 | 0.0124 | 0.0526 |

| **Table S7.** Propensity score matching results for MID3 | | | | | | |
| --- | --- | --- | --- | --- | --- | --- |
|  | **Means Treated** | **Means Control** | **Std. Mean Diff.** | **Var. Ratio** | **eCDF Mean** | **eCDF Max** |
| Distance | 0.1151 | 0.1149 | 0.0035 | 1.0186 | 0.001 | 0.0148 |
| Male | 0.6267 | 0.6073 | 0.0401 |  | 0.0194 | 0.0194 |
| Female | 0.3733 | 0.3927 | -0.0401 |  | 0.0194 | 0.0194 |
| Dutch | 0.5788 | 0.5856 | -0.0139 |  | 0.0068 | 0.0068 |
| Non-Dutch European | 0.0616 | 0.0742 | -0.0522 |  | 0.0126 | 0.0126 |
| Non-European  Caribbean | 0.113 | 0.1187 | -0.018 |  | 0.0057 | 0.0057 |
| Non-European  Moroccan/Turkish | 0.1199 | 0.1221 | -0.007 |  | 0.0023 | 0.0023 |
| Non-European  African | 0.0616 | 0.0479 | 0.057 |  | 0.0137 | 0.0137 |
| Non-European  Indonesian | 0.0651 | 0.0514 | 0.0555 |  | 0.0137 | 0.0137 |
| Primary or lower maternal education | 0.0925 | 0.0902 | 0.0079 |  | 0.0023 | 0.0023 |
| Secondary maternal education | 0.4726 | 0.5034 | -0.0617 |  | 0.0308 | 0.0308 |
| Higher maternal education | 0.4349 | 0.4064 | 0.0576 |  | 0.0285 | 0.0285 |
| Low income (< 1200 €/month) | 0.2021 | 0.2009 | 0.0028 |  | 0.0011 | 0.0011 |
| Middle (1200-2000 €/month) | 0.1404 | 0.1324 | 0.023 |  | 0.008 | 0.008 |
| Higher (>2000 €/month) | 0.6575 | 0.6667 | -0.0192 |  | 0.0091 | 0.0091 |
| Age at T1 | 1.5338 | 1.5343 | -0.0071 | 0.7808 | 0.0072 | 0.0388 |

| **Table S8.** Propensity score matching results for MID4 | | | | | | |
| --- | --- | --- | --- | --- | --- | --- |
|  | **Means Treated** | **Means Control** | **Std. Mean Diff.** | **Var. Ratio** | **eCDF Mean** | **eCDF Max** |
| Distance | 0.0888 | 0.0886 | 0.0035 | 1.0279 | 0.0006 | 0.0087 |
| Male | 0.6288 | 0.6215 | 0.0151 |  | 0.0073 | 0.0073 |
| Female | 0.3712 | 0.3785 | -0.0151 |  | 0.0073 | 0.0073 |
| Dutch | 0.5721 | 0.575 | -0.0059 |  | 0.0029 | 0.0029 |
| Non-Dutch European | 0.0699 | 0.067 | 0.0114 |  | 0.0029 | 0.0029 |
| Non-European  Caribbean | 0.1179 | 0.1266 | -0.0271 |  | 0.0087 | 0.0087 |
| Non-European  Moroccan/Turkish | 0.1048 | 0.0975 | 0.0238 |  | 0.0073 | 0.0073 |
| Non-European  African | 0.0699 | 0.0757 | -0.0228 |  | 0.0058 | 0.0058 |
| Non-European  Indonesian | 0.0655 | 0.0582 | 0.0294 |  | 0.0073 | 0.0073 |
| Primary or lower maternal education | 0.0961 | 0.099 | -0.0099 |  | 0.0029 | 0.0029 |
| Secondary maternal education | 0.4323 | 0.4265 | 0.0118 |  | 0.0058 | 0.0058 |
| Higher maternal education | 0.4716 | 0.4745 | -0.0058 |  | 0.0029 | 0.0029 |
| Low income (< 1200 €/month) | 0.2052 | 0.2038 | 0.0036 |  | 0.0015 | 0.0015 |
| Middle (1200-2000 €/month) | 0.131 | 0.1441 | -0.0388 |  | 0.0131 | 0.0131 |
| Higher (>2000 €/month) | 0.6638 | 0.6521 | 0.0246 |  | 0.0116 | 0.0116 |
| Age at T1 | 1.5364 | 1.5344 | 0.025 | 1.002 | 0.0047 | 0.0247 |

| **Table S9.** Number of MID participants and matched controls per MID operationalization. | | | |
| --- | --- | --- | --- |
|  | **MID**  **n (%^a^)** | **Control**  **n (%^a^)** | **Total**  **n (%^a^)** |
| **MID1 (IQ ≤ 75)** | 115 (2.5%) | 345 (7.5%) | 460 (9.9%) |
| **MID2 (IQ ≤ 75 + Conc., Soc., or Prac.)** | 82 (1.8) | 246 (5.3) | 328 (7.1) |
| **MID3 (IQ ≤ 85 + Conc., Soc., or Prac.)** | 408 (8.8%) | 1224 (26.4%) | 1632 (35.1%) |
| **MID4 (IQ ≤ 85 + Conc.)** | 234 (5.0%) | 702 (15.1%) | 936 (20.1%) |
| **Any** | 441 (9.5%) | 1406 (30.3%) | 1847 (39.8%) |
| *Note:* MID = Mild Intellectual Disability group; Control = Matched control for the MID group; Conc. = Conceptual functioning problems; Soc. = Social functioning problems; Prac. = Practical functioning problems.  ^a^ Refers to the proportion of the Generation R study sample (n = 4643) (Figure S1). | | | |

| **Table 10.** Distribution of adaptive functioning indicators across MID definitions | | | | |
| --- | --- | --- | --- | --- |
|  | **n cases** | **Conceptual functioning impairment, n (%)** | **Social functioning z-score, median (IQR) [range]** | **Practical functioning z-score, median (IQR) [range]** |
| **MID1 (IQ ≤ 75)** | 115 | 80 (69.6%) | 0.90 (0.04–1.75)  [-1.09–6.30] | 0.37 (-0.63–1.38)  [-1.13–3.89] |
| **MID2 (IQ ≤ 75 + Conc., Soc., or Prac.)** | 82 | 80 (97.6%) | 1.18 (0.04–2.03)  [-1.09–6.30] | 0.37 (-0.63–1.38)  [-1.13–3.89] |
| **MID3 (IQ ≤ 85 + Conc., Soc., or Prac.)** | 408 | 234 (57.3%) | 1.18 (0.04–2.32)  [-1.09–6.87] | 0.88 (-0.13–2.39)  [-1.13–4.40] |
| **MID4 (IQ ≤ 85 + Conc.)** | 234 | 234 (100%) | 0.61 (0.04–1.75) [-1.09–6.30] | 0.37 (-0.63–0.88) [-1.13–3.89] |
| *Note:* MID = Mild Intellectual Disability group; Conc. = Conceptual functioning problems; Soc. = Social functioning problems; Prac. = Practical functioning problems. | | | | |

| **Table S11.** Association between MID4 status and psychopathology domains over time. | | | | | | |
| --- | --- | --- | --- | --- | --- | --- |
|  | **MID4 (IQ ≤ 85 + Conc.)** | | | | | |
|  | **Main effect** | | | **Age Interaction** | | |
|  | **Std β (SE)** | ***p*** | ***p*FDR** | **Std β (SE)** | ***p*** | ***p*FDR** |
| **Somatic Problems** | 0.11 (0.06) | .08 | .10 | 0.01 (0.02) | .66 | .75 |
| **Picky eating** | 0.23 (0.06) | <.001 | <.001 | 0.01 (0.02) | .41 | .56 |
| **ASD** | 0.27 (0.06) | <.001 | <.001 | 0.08 (0.02) | <.001 | .001 |
| Main effect at T1 |  |  |  | 0.04 (0.11) | .65 | .75 |
| **Sleep Problems** | 0.08 (0.06) | .19 | .25 | 0.03 (0.02) | .16 | .32 |
| **Affective** | 0.19 (0.05) | <.001 | .001 | 0.00 (0.01) | .42 | .56 |
| **Anxiety** | 0.24 (0.06) | .001 | .002 | 0.01 (0.01) | .16 | .32 |
| **ADHD** | 0.40 (0.05) | <.001 | <.001 | 0.03 (0.01) | <.001 | <.001 |
| Main effect at T1 |  |  |  | 0.13 (0.08) | .70 | .75 |
| **ODD** | 0.20 (0.05) | <.001 | <.001 | 0.01 (0.01) | .09 | .21 |
| *Note*: Linear mixed-effects models were used to test the associations between MID status and repeated psychopathology domains. Affective, anxiety, ADHD, and ODD problems were analysed from T1 to T5, whereas somatic problems, picky eating, ASD problems, and sleep problems were analysed from T1 to T3. Standardized effect estimates including the main effect and interaction effect(*β*) (Symptoms change; interaction of MID*age), as well as standard errors (SE), *p*, and *p*FDR values are shown. Conc. = Conceptual functioning problems; MID = Mild Intellectual Disability group; IQ = Intelligence quotient based on the WISC-V; ASD = autism spectrum disorder problems. ADHD = Attention-deficit/hyperactivity problems; ODD = Oppositional defiant problems. | | | | | | |

**References**

1.	Achenbach TM (2011) Child Behavior Checklist. In: Kreutzer JS, DeLuca J, Caplan B (eds) Encyclopedia of Clinical Neuropsychology. Springer, New York, NY, pp 546–552

2.	Constantino JN, Davis SA, Todd RD, et al (2003) Validation of a Brief Quantitative Measure of Autistic Traits: Comparison of the Social Responsiveness Scale with the Autism Diagnostic Interview-Revised. J Autism Dev Disord 33:427–433. https://doi.org/10.1023/A:1025014929212

3.	Gioia GA, Isquith PK, Guy SC, Kenworthy L (2000) TEST REVIEW Behavior Rating Inventory of Executive Function. Child Neuropsychol 6:235–238. https://doi.org/10.1076/chin.6.3.235.3152

4.	Brown TA, Moore MT (2012) Confirmatory factor analysis. In: Handbook of structural equation modeling. The Guilford Press, New York, NY, US, pp 361–379

5.	Rosseel Y (2012) lavaan: An R Package for Structural Equation Modeling. J Stat Softw 48:1–36. https://doi.org/10.18637/jss.v048.i02

6.	(2019) ASEBA - PC - ASEBA. https://aseba.org/aseba-pc/. Accessed 15 Dec 2023

7.	Mulder TA, Kocevska D, Muetzel RL, et al (2019) Childhood sleep disturbances and white matter microstructure in preadolescence. J Child Psychol Psychiatry 60:1242–1250. https://doi.org/10.1111/jcpp.13085

8.	Cardona Cano S, Tiemeier H, Van Hoeken D, et al (2015) Trajectories of picky eating during childhood: A general population study. Int J Eat Disord 48:570–579. https://doi.org/10.1002/eat.22384

9.	Kooijman MN, Kruithof CJ, van Duijn CM, et al (2016) The Generation R Study: design and cohort update 2017. Eur J Epidemiol 31:1243–1264. https://doi.org/10.1007/s10654-016-0224-9
